# Comparison of the Opinions of Adolescents With Different Orthodontic Treatment Needs

**DOI:** 10.1002/cre2.944

**Published:** 2024-08-28

**Authors:** Larisa Krekmanova, Shams Shakrchi, Amina Gicic, Julia Naoumova

**Affiliations:** ^1^ Department of Paediatric Dentistry, Institute of Odontology, Sahlgrenska Academy University of Gothenburg Gothenburg Sweden; ^2^ Public Dental Service Region Värmland Karlstad Sweden; ^3^ Public Dental Service Västra Götaland County Gothenburg Sweden; ^4^ Specialist Clinic for Orthodontics Public Dental Service Region Västra Götaland Gothenburg Sweden; ^5^ Department of Orthodontics, Institute of Odontology, Sahlgrenska Academy University of Gothenburg Gothenburg Sweden

**Keywords:** adolescents, IOTN, oral health‐related quality of life, orthodontic treatment, PROM

## Abstract

**Background:**

The aim of this study was to compare oral health‐related quality of life (OHRQoL) among adolescents granted (G) versus not granted (NG) publicly funded orthodontic treatment.

**Materials and Methods:**

Adolescents aged 15–20 years who were granted versus not granted publicly funded orthodontic treatment responded to a web‐based survey, assessing OHRQoL in relation to functional impact, psychological impact, and motivators for orthodontic treatment. Before the survey, pretesting of the questionnaire was conducted to ensure its reliability. Differences between the groups were tested using the chi‐squared and Mann–Whitney *U*‐tests. Reliability was assessed using Cohen's *κ* and Pearson's correlation coefficient during the pretest phase.

**Results:**

One hundred and forty patients, equally distributed between a G and an NG group, responded to the survey. Gender, age, and demographic distribution were comparable in both groups. All respondents expressed a high subjective treatment need and similar answers regarding functional aspects. Orthodontic treatment motivators (G: 86.2% and NG: 94.7%, *p* = 0.443) were primarily related to improved self‐esteem, overall well‐being, facial appearance, and being able to laugh without embarrassment. The NG group expressed a more negative OHRQoL impact due to the appearance of their teeth compared with the G group (*p* < 0.001) and a negative impact caused by the position of their teeth (*p* < 0.001).

**Conclusions:**

Orthodontic treatment need indices should aim to reinforce subjective measures as adolescents express similar motivators for orthodontic treatment, regardless of the clinician's objectively based decision about treatment need.

## 1 Introduction

Oral health‐related quality of life (OHRQoL) refers to the subjectively experienced oral status correlated to general health and overall well‐being. This instrument focuses not only on the presence or absence of oral pathology but also considers how oral conditions can affect the functional, psychological, and social aspects of daily life. Certain oral conditions that affect the ability to chew, speak, and smile may distress individuals significantly during social interactions (Sischo and Broder 2011).

Over the decades, treatment need indices have been developed to assist professionals in making decisions regarding the severity of patients' morphological and aesthetic deviations (Brook and Shaw 1989). However, the criteria for granting orthodontic treatment may vary significantly due to the specific social and economic systems on which dental care systems in different countries are based (Winkelmann, Gómez, and van Ginneken 2022). There are indications that orthodontic treatment demand among young people has increased, possibly influenced by the increased focus on facial appearance. For this reason, this issue requires professional acknowledgment and attention (Thilander and Myrberg 1973; Borzabadi‐Farahani 2011).

In countries where the government subsidizes dental health services, as in Sweden, orthodontic treatment need indices are used to allocate resources in a measurable manner. Each index has cut‐off points for eligibility to receive publicly funded treatment. Depending on the available resources and perceived need, the cut‐off points can be modified (Borzabadi‐Farahani 2012). In Sweden, the Index of Orthodontic Treatment Need (IOTN), with regional adaptations, is the most frequently used index. However, *The Swedish Agency for Health Technology Assessment and Assessment of Social Services* (Swedish Council on Technology Assessment [SBU]) reported in 2005 that there is insufficient evidence to support the assumption that patients with a higher IOTN score are at greater risk of oral conditions and, consequently, have a greater objective treatment need (SBU 2005). The predictive validity of identifying individuals with a higher risk of oral health problems, using only a *morphological treatment need index*, has also been questioned due to the lack of a sufficiently subjective dimension (SBU 2005; Mohlin and Kurol 2003). Furthermore, the SBU report concludes that the predominant motivator for seeking orthodontic treatment is the perception of oral aesthetics (SBU 2005). Researchers also indicate other internal and external factors that motivate individuals to undergo orthodontic treatment, such as gender, age, social circumstances, bullying, the severity of the malocclusion, and oral function (Bauss and Vassis 2023; Feldens et al. 2015; Trulsson et al. 2022). Malocclusion in young patients has been reported to affect their self‐esteem negatively and lead to strategies such as avoiding smiling (Trulsson et al. 2022; Bayat 2016). These results were recently confirmed in a systematic review (Dimberg, Arnrup, and Bondemark 2015). There is a lack of knowledge regarding the treatment demands of adolescents in relation to the actual availability of orthodontic treatment covered by the health care system and the professional assessment based on the applied indices.

Therefore, the aim was to compare the OHRQoL between adolescents who have received orthodontic treatment covered by the national public health care system and those who have not been granted such treatment.

The hypothesis was that there would be no differences in OHRQoL between the adolescents granted orthodontic treatment covered by the national public healthcare system and those who were not granted such treatment.

## 2 Subjects and Methods

This study was an observational web‐based survey conducted in Gothenburg, Sweden, between June and December 2021. The protocol of the study received approval by the research ethics committee of the Swedish Ethical Review Authority (Dnr 2021‐01867).

### 2.1 Patients

The study sample comprised adolescents who were referred from 22 public dental clinics in Gothenburg to the Clinic of Orthodontics, Gothenburg, Sweden, either for orthodontic treatment or a second assessment of orthodontic treatment need. The participants were consecutively recruited by seventeen consulting orthodontists and divided into two groups: a group that was granted (G) orthodontic treatment and a group that was not granted (NG) orthodontic treatment.

*The G orthodontic treatment group* consisted of patients between 15 and 20 years of age who had been approved for treatment covered by the public healthcare system. All patients in this group had malocclusions falling within IOTN indices 4 and 5, with a few in index 3, making them eligible for free orthodontic treatment in Region Västra Götaland.

*The NG group* consisted of patients between 15 and 20 years of age who had a subjective orthodontic treatment need but were denied such treatment by the consulting orthodontist. This decision was taken as the malocclusion was assessed not to be severe enough to qualify for free orthodontic treatment according to the guidelines in Region Västra Götaland, which are partly based on the dental health component (DHC) of the IOTN (Brook and Shaw 1989) and partly using a local extended modified version of the aesthetic component (AC). Since these patients had a subjective orthodontic treatment need, they were referred to the specialist clinic in Gothenburg, Sweden, for a second opinion. A group of five appointed orthodontists independently assessed the malocclusion based on a standardized template of intraoral and extraoral photographs and the outcome was determined by majority decision. Based on this assessment, the patients in the group would either receive or not receive orthodontic treatment covered by the public healthcare system.

### 2.2 Method

During the study period, phone numbers for all patients meeting the inclusion criteria were obtained from dental records. The participants received a text message containing written information about the study and were required to provide digital consent before accessing the questionnaire.

#### 2.2.1 Questionnaire

An expert panel consisting of senior researchers from two disciplines. orthodontics and pedodontics, complied the items of the questionnaire resulting in 22 close‐ended questions to assess three distinct domains: *functional impact, psychological impact*, and *motivators for orthodontic treatment*, after which the content validity was assessed. Thus, 12 validated questions from the short‐form Child Perceptions Questionnaire 11–14 (Dimberg et al. 2019) were adopted. Further two questions were modified from another validated survey (Bayat 2016). Two questions were constructed for this survey. The remaining questions were demographic in nature.

Among the questions, 11 had multiple‐choice response options: “never,” “once a month,” “once a week,” or “daily.” Additionally, the questionnaire contained two questions requiring the participants to rate their self‐esteem and perceived orthodontic treatment need using a 10‐point Likert‐like scale ranging from 0 (*not at all*) to 10 (*very much*). For all questions, only response was allowed. Multiple responses were possible for two questions: Q18 and Q20 (File S1). All questions were mandatory, and response editing was not possible.

The questionnaire was distributed continuously over a 6‐month period using Webropol, an online survey platform. Each participant received two reminders: the first one 2 weeks after the initial text message and the second reminder 1 week after the first.

#### 2.2.2 Pretesting

Before pretesting, face validity of the questions was assessed by five dental students, after which two questions (Q18 and Q21) were modified to increase comprehension.

Then, pretesting of the questionnaire was conducted to assess its reliability. Thirteen dental students from the Institute of Odontology at the University of Gothenburg, Sweden (aged 19–20 years), as well as 10 adolescents (aged 15–18 years), participated in the pretesting. Ten days later, the participants were asked to respond to the same questions again.

### 2.3 Statistics

The sample size was based on a previous study calculation that two scale standard deviations between the G and NG groups were considered a significant clinical difference (Göranson et al. 2021). The sample size analysis yielded that a minimum of 63 individuals in each group was needed to acquire a power of 80%, with a significance level of 0.05. The patient number in each group was increased to 70 to allow for incomplete questionnaire answers.

The statistical analysis was conducted using the IBM SPSS Statistics version 28.0.1 software (IBM Corp, Armonk, NY, USA). Cohen's *κ* was used to assess reliability, while Pearson's correlation coefficient was used to assess the correlation between the test and the retest. Descriptive statistics were calculated for demographic data on the group level. The chi‐squared test was applied to analyze the observed results in relation to the expected results. The Mann–Whitney *U*‐test was used for comparison of the two independent samples.

The questions with multiple choice response options were dichotomized as *rarely* = “never” and “once a month” and *often* = “daily” and “once a week.” The response options to the question, “If you want braces, give a reason why,” were divided into five categories: *Belonging* (“My parents think I should,” “My friends have it, I want it too,” “Easier to get along with others,” “Easier to get a romantic partner” *a boyfriend/girlfriend?*, “Straight teeth are expected in society/social media”), *Psychosocial well‐being* (“To feel better,” “To be able to smile/laugh without feeling embarrassed,” “Others will stop teasing me,” “To feel confident”), *Financial and future aspects* (“Get better work in the future,” “Will not regret it in the future,” “Because it's free”), *Esthetic* = “To improve my appearance”), and *Oral health* (“An investment in my oral health”).

The response options to the question, “Those who think I need braces,” that is, “Family,” “Peers,” and “Partner,” were grouped into a new category called *Family and relatives*.

The response options to the statement, “I would consider paying for braces if the treatment were not free,” were dichotomized into > or < €400.

## 3 Results

The reliability of the questionnaire was considered high with Cohen's weighted *κ* at 0.800 (95% CI: 0.734, 0.866) and Pearson's correlation coefficient at 0.852.

The questionnaire was continuously distributed until 70 questionnaires were completed in each group. The response rate in the G group was 27.0% (70/255), compared with 51.0% (70/136) in the NG group. In total, the G group received 637 mailings, whereas the NG group received 293 mailings, including reminders. Both groups showed similar distributions regarding gender, age, and demographic area (Table 1).

**Table 1:** Demographic data for the participants (*N* = 140).

Variables	G group (*N* = 70), No. (%)	NG group (*N* = 70), No. (%)
Gender		
Female	39 (55.7)	37 (52.9)
Male	30 (42.9)	31 (44.3)
Other	1 (1.4)	2 (2.8)
Age (years)		
15	16 (22.9)	17 (24.3)
16	13 (18.6)	13 (18.5)
17	19 (27.1)	17 (24.3)
18	15 (21.4)	10 (14.3)
19	5 (7.1)	7 (10.0)
20	2 (2.9)	6 (8.6)
Region		
Northern Gothenburg	24 (34.2)	27 (38.5)
Southern Gothenburg	37 (52.9)	37 (52.9)
Uncertain	9 (12.9)	6 (8.6)

**Figure 1:**
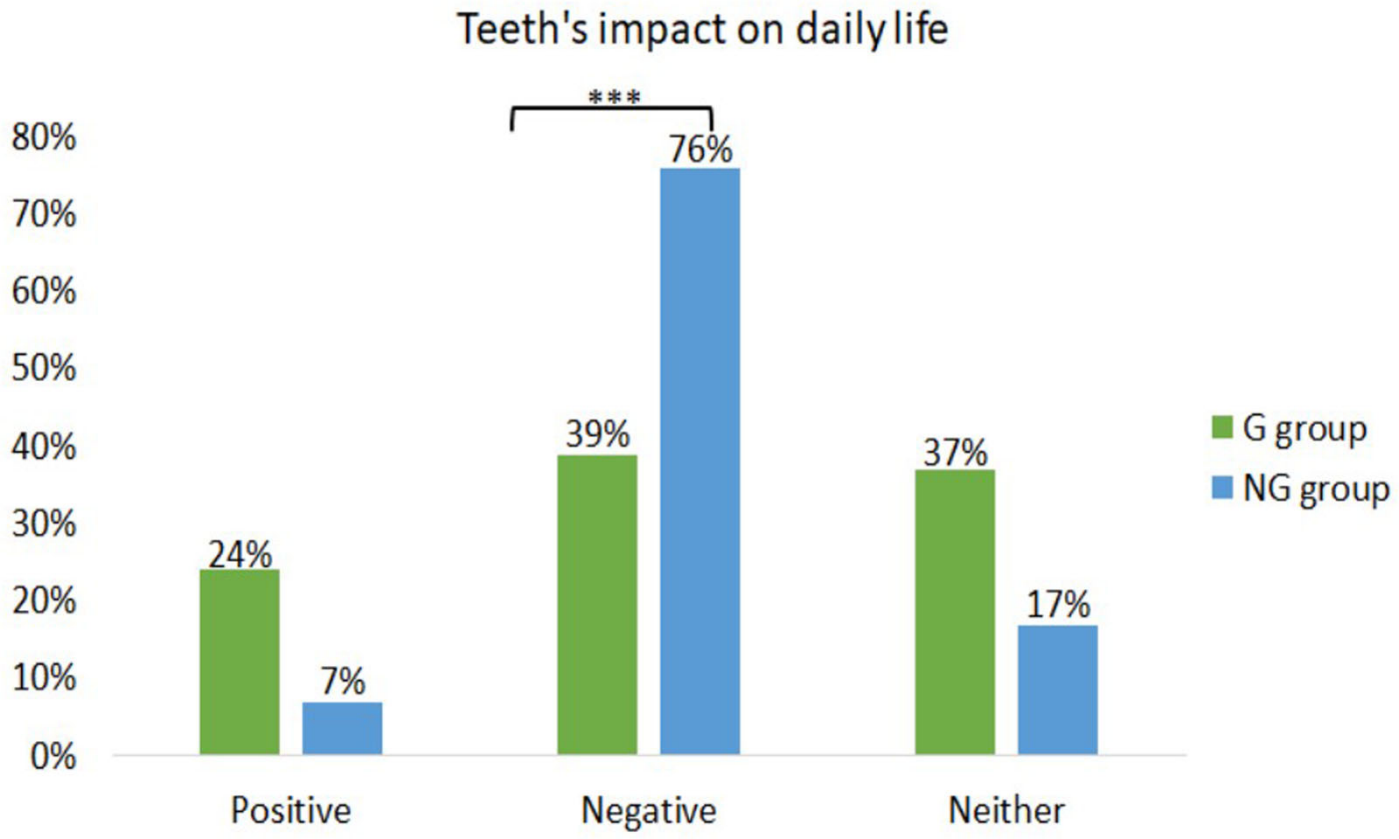
Percentage of responses indicating how the teeth impact daily life among the G and NG group individuals. ***p < 0.001.

The survey revealed that a significantly larger proportion of individuals in the NG group than in the G group (*p* < 0.001) reported that their teeth had a negative impact on their lives (Figure 1).

### 3.1 Functional Impact

Similar responses to function‐related questions were observed in both the G group and the NG group, with the majority reporting no significant difficulties in oral functions such as chewing, biting, or speaking. However, a nonstatistically significant difference was found, with 70.0% of the NG group expressing more frequent food impaction compared with 57.1% in the G group. Similarly, a higher percentage of individuals in the G group (17.1%) expressed more frequent speech difficulties due to malocclusion, compared with the NG group (12.9%).

### 3.2 Psychosocial Impact

**Figure 2:**
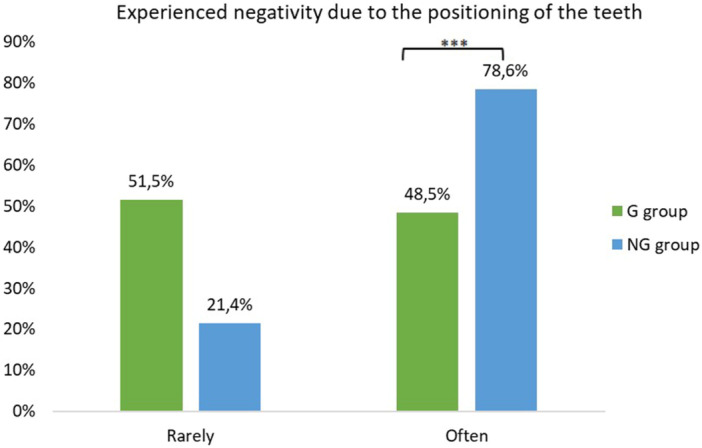
Response percentages regarding the experienced negativity due to the positioning of the teeth, among the G and NG group individuals. ***p < 0.001.

Participants in the G group experienced greater difficulties when smiling or laughing in different social settings, compared with the NG group (57.1% and 32.8%, respectively, *p* = 0.004). However, participants in the NG group engaged in behaviors such as covering their mouth while laughing more often than participants in the G group (64.3% and 44.3%, respectively, *p* = 0.018). One‐third of the participants in the NG group reported being bullied more often than those in the G group (30.0% and 15.7%, respectively, *p* = 0.044). Additionally, the NG group experienced significantly more negativity due to the positioning of their teeth compared with the G group (78.6% and 48.5%, respectively, *p* < 0.001) (Figure 2).

**Figure 3:**
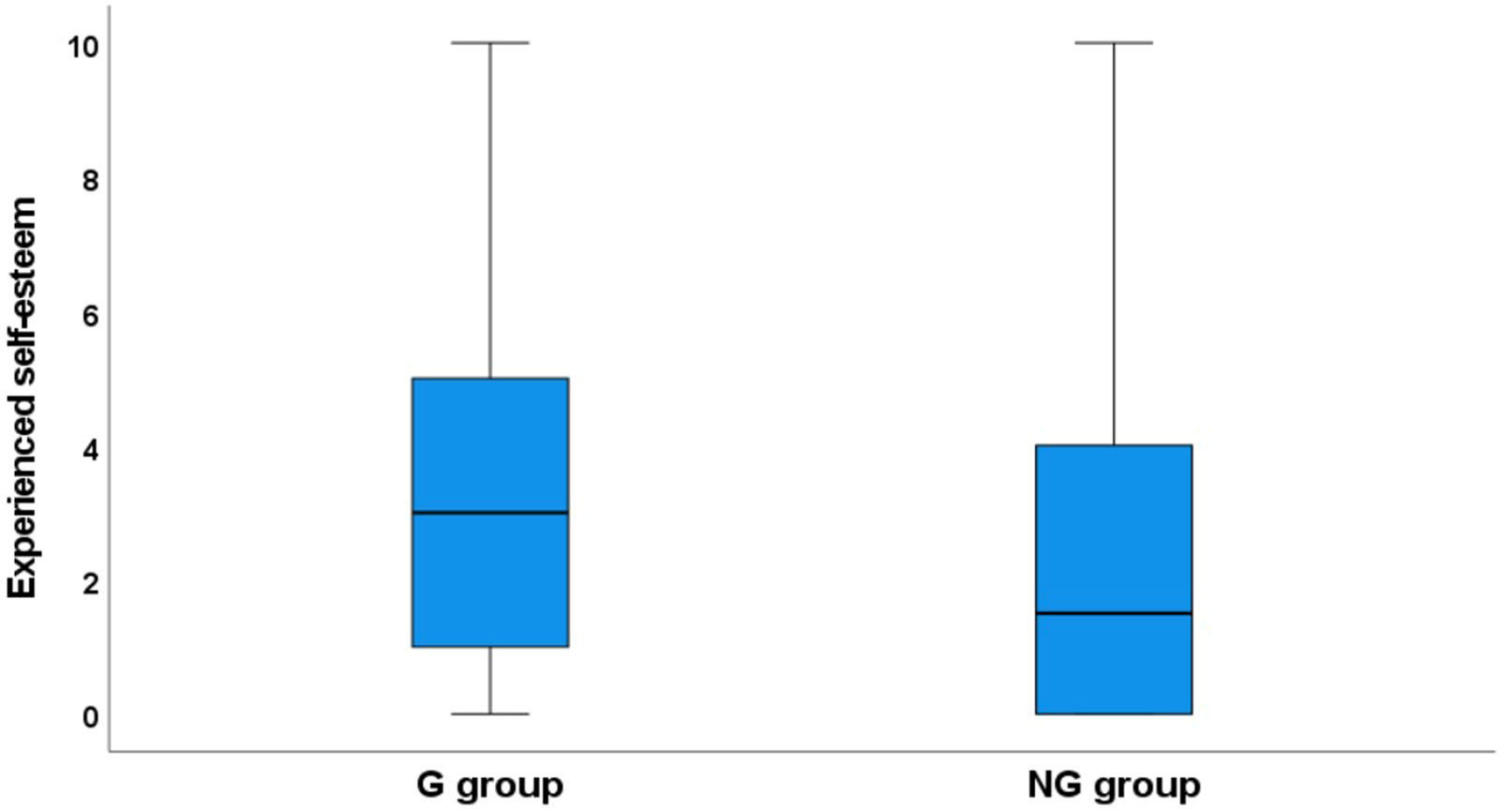
Box plot displaying experienced self‐esteem (0–10: 0 = *no treatment need*, 10 = *maximum treatment need*) as reported by the G and NG groups, *p* = 0.193.

Similar levels of low dental self‐esteem were observed in both groups *p* = 0.193 (Figure 3).

### 3.3 Motivators for Treatment

**Figure 4:**
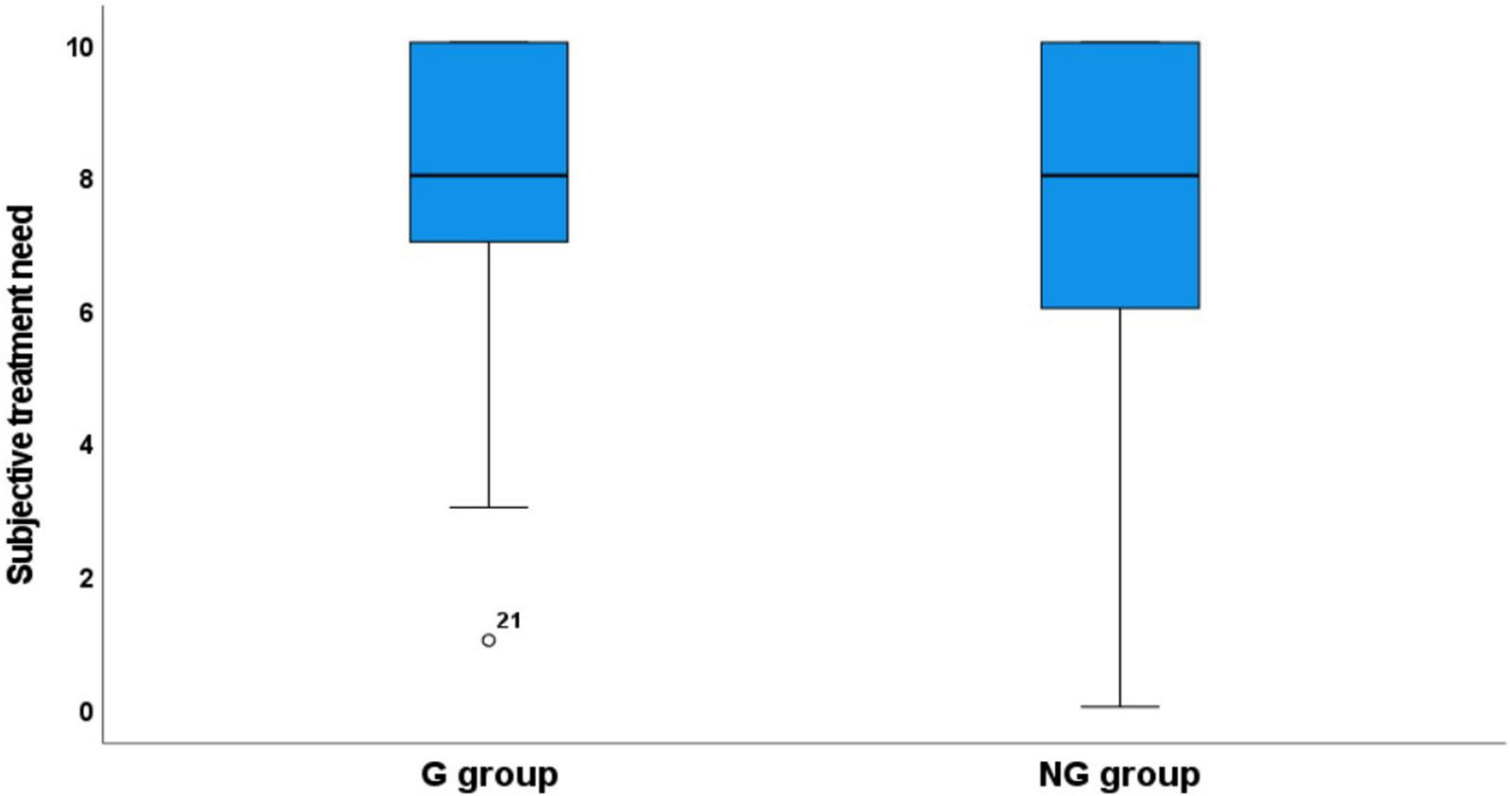
Box plot displaying the distribution of subjective treatment need (0–10: 0 = *no treatment need*, 10 = *maximum treatment need*) among individuals in the G and NG groups, *p* = 0.549.

Regardless of group affiliation and objective treatment need, the respondents experienced high subjective treatment needs (Figure 4).

All individuals in the NG group expressed a desire for orthodontic treatment, compared with 98.2% in the G group. Motivators for orthodontic treatment in both groups (G: 86.2 and NG: 94.7%, *p* = 0.443) were primarily to improve self‐esteem, overall well‐being, facial appearance, and being able to laugh without feeling embarrassed.

Other frequently cited motivators in both groups were “avoiding future regrets” (G: 63.1% and NG: 44.7%, *p* = 0.6849) and “enhancing employment opportunities” (G: 15.4% and NG: 5.3%, *p* = 0.865). Open‐ended questions regarding subjective treatment needs were responded to by both groups as a desire to improve conditions for oral and functional health. The need for treatment was primarily expressed by the respondents themselves or their family members. A notable difference was seen between the G and NG groups whether the dentist had suggested the need for orthodontic treatment (G: 64.3% and NG: 13.2%, *p* = < 0.001). When asked about their willingness to pay for treatment, most respondents were willing to pay less than €400 (G: 72.9% and NG: 55.3%, *p* = 0.288). The individuals in the NG group tended to be prepared to pay more than €400 (44.7% and 27.1%, respectively, *p* = 0.425).

## 4 Discussion

The assessment of orthodontic treatment needs is often motivated by and based on various indices, without fully considering the patient's perspective, thus not considering subjective experiences and wishes (Tsichlaki and O'Brien 2014; de Oliveira et al. 2008). The objective of this study was therefore to explore the QoL of adolescents who have been granted versus not granted orthodontic treatment covered by the national public healthcare system. The main finding showed that a significantly larger number of patients in the NG group felt that their teeth impacted their OHRQoL negatively.

Several studies using generic measures of OHRQoL have shown that malocclusion predominantly affects the emotional and social dimensions of young people's everyday life (Bayat 2016; Dimberg, Arnrup, and Bondemark 2015; Dimberg et al. 2019; Meade and Dowswell 2016; Liu, McGrath, and Hägg 2009). In addition, orthodontic treatment for different types of malocclusions has been shown to improve OHRQoL of life (Palomares et al. 2012; Feu et al. 2013). Furthermore, previous studies have shown that children younger than 13 years of age have difficulties discussing aesthetics in relation to orthodontics (Meade and Dowswell 2016). Therefore, this study focused on adolescents aged 15–20 years, who responded to a web‐based survey containing three domains: functional impact, psychological impact, and motivators for orthodontic treatment.

While some have reported that young patients usually seek orthodontic treatment for functional and aesthetic concerns (de Oliveira et al. 2008), others report that the dominant reason is improved appearance rather than improved function (Borzabadi‐Farahani 2012; Trulsson et al. 2022; Bayat 2016; Wędrychowska‐Szulc and Syryńska 2010; Patel et al. 2016). In the present study, no oral function difficulties were reported in both groups, which is in contrast to previous findings (Borzabadi‐Farahani 2012; Henrikson, Ekberg, and Nilner 2009; Alshammari et al. 2022). Various orthodontic treatment need indices have been developed to quantify malocclusion severity and standardize the assessment of treatment needs in an objective manner (SBU 2005). Two widely used indices are the IOTN and the Index of Complexity, Outcome, and Need (ICON) (Brook and Shaw 1989; Daniels and Richmond 2000). Although both indices include an AC, they are still clinician‐based evaluations, measuring deviations from normal occlusion rather than perceived treatment need. However, perceived treatment need does not always reflect the normative treatment needs, which is why orthodontic treatment need indices should be reinforced with OHRQoL measures, such as the oral aesthetic subjective impact scale (Ghijselings et al. 2014). The findings from this study support this, as participants in both groups expressed their social deficiencies differently, either covering their mouth while laughing (NG) or avoiding smiling (G). Furthermore, the NG group reported more often being bullied and felt greater negativity due to the positioning of their teeth.

Adolescence is a vulnerable and critical time for identity formation regarding physical, psychological, and social changes (Meade and Dowswell 2016). In the current study, the respondents rated their self‐confidence equally low in both groups. This can affect the individual's social interaction and overall well‐being, thus reducing their OHRQoL. Insecurity may manifest itself as problems with smiling and laughing in social settings, leading to the development of coping strategies such as covering the teeth, altering smiles, or withdrawing from social groups (Trulsson et al. 2022; Bayat 2016; Patel et al. 2016). Furthermore, certain occlusal conditions have been associated with bullying, particularly among adolescents (Seehra et al. 2011). The motivators for wishing to undergo orthodontic treatment in this study were to improve self‐esteem, well‐being, facial appearance, and the ability to laugh without feeling embarrassed. These motivators were reported to a similar extent in both groups. Young people often undergo orthodontic treatment on the assumption that it will help resolve their social issues since improved aesthetics via orthodontic treatment is thought to enhance psychosocial well‐being (Borzabadi‐Farahani 2012; Bayat 2016; Benson, Javidi, and DiBiase 2015).

Various factors motivate patients to undergo orthodontic treatment, and although adolescents may not be fully aware of it, their motivation is influenced by external factors such as social norms and beauty culture in their reference groups and society (Trulsson et al. 2022). This is not surprising, as smiling is important in social interactions and communication and for self‐perception (Kokich, Asuman Kiyak, and Shapiro 1999; Kokich, Kokich, and Kiyak 2006). A strong correlation has been found between smile attractiveness and facial attractiveness, as well as self‐perception and self‐esteem (Godinho, Gonçalves, and Jardim 2020). Furthermore, adolescents with a normal dental appearance are not only perceived as more attractive but also as more desirable as friends, more socially accepted by peers, teachers, and others, and as more intelligent (Trulsson et al. 2022). With the increased influence of social media, the demand for aesthetic dentistry has grown (SBU 2005). Greater dissatisfaction with their dental appearance is often seen among younger individuals (Trulsson et al. 2022; Deli et al. 2012; Christopherson, Briskie, and Inglehart 2009) and more common among girls than boys (Deli et al. 2012; Christopherson, Briskie, and Inglehart 2009).

The response rate in the current study was higher among the adolescents in the NG group. A possible reason for this outcome could be a greater need to have their voices heard, considering they were not granted publicly funded treatment. Orthodontic treatment need indices are used in many countries, despite their being based on consensus rather than research evidence (SBU 2005). As mentioned previously, these indices should incorporate the patient's subjective need for treatment. However, it might be difficult to determine the lowest subjective treatment need score that would grant an individual publicly funded treatment. Therefore, future studies should use patient‐reported outcome measures to assess changes in social well‐being before and after treatment to establish an evidence‐based scoring system for orthodontic treatment need indices.

### 4.1 Limitations

The current study did not consider the severity of the malocclusion, the orthodontic treatment need scoring, or the socioeconomic background of the patients, which could be a potential confounding factor influencing the responses. In addition, the patient's awareness of whether they are prioritized or not for publicly funded treatment might have affected their answers, possibly explaining the more optimistic responses in the G group. Furthermore, the NG group was aware of their non‐priority status, which may have reinforced the negative feelings they already associated with the appearance of their teeth. A further limitation is the lack of external validity of the questionnaire. The questionnaire needs to be applied to different populations over time. Thus, the results of the study could yet not be generalized.

### 4.2 Generalizability

Socioeconomic background may affect a person's perception of their malocclusion (Reichmuth et al. 2005). In the current study, the patient *catchment* area for the Specialty Clinic of Orthodontics comprises 22 public dental clinics in Gothenburg, with approximately 550,000 inhabitants exhibiting variations regarding living conditions and socioeconomic status. Therefore, the similar distribution of respondents in the G group and the NG group across the various city areas suggests generalizability of the results. Therefore, the obtained results can only be generalized to countries where the government subsidizes dental health services and utilizes the IOTN, with the same cut‐off points for adolescents in the same age interval as the present study.

## 5 Conclusions

Regardless of the clinician's objectively based decision for treatment need, the respondents expressed similar motivators for orthodontic treatment. However, the patients not prioritized for publicly funded treatment reported more experienced negativity due to the positioning of their teeth with a negative impact on their OHRQoL. Therefore, orthodontic treatment need indices should aim to reinforce subjective measures.

## Author Contributions

Conception and design of the study: J.N. and L.K. Data collection: S.S. and A.G. Interpretation of data: L.K., J.N., S.S., and A.G. Statistics: S.S. and A.G. Manuscript writing: L.K., J.N., S.S., and A.G. All authors read and approved the final manuscript.

## Ethics Statement

The protocol of the study was approved by the research ethics committee of the Swedish Ethical Review Authority (Reg. No: 2021‐01867).

## Consent

Informed consent was obtained in writing from the study participants.

## Conflicts of Interest

The authors declare no conflicts of interest.

## Supporting information

Supporting information.

## Data Availability

The data on which this article is based will be shared upon reasonable request to the corresponding author.
