# Supplementary material for: Comparison of the Opinions of Adolescents With Different Orthodontic Treatment Needs
Source: Clin Exp Dent Res. 2024 Aug 28;10(4):e944. doi: 10.1002/cre2.944 (PMC11358389; doi:10.1002/cre2.944)
Supplement: Supplementary file 1 — Supporting information. [file CRE2-10-e944-s001.docx]

PROJECT: How I experience my teeth

If you are 15-20 years old and have had your teeth examined by an orthodontic specialist, how do you experience your teeth?

Please check the appropriate box.

1. I am a ❑ girl ❑ boy
2. This year I will turn ❑ ❑ ❑ ❑ ❑ ❑

15 years 16 years 17 years 18 years 19 years 20 years

1. I go to the dentist at this public dental care service:

❑ Angered ❑ Askim ❑ Axel

❑ Bergsjön ❑ Biskopsgården ❑ Björkekärr

❑ Frölunda Kulturhus ❑ Gamlestaden Kulan ❑ Gibraltar

❑ Guldheden ❑ Hovås ❑ Hjällbo

❑ Järntorget ❑ Kortedala ❑ Kvillebäcken

❑ Kärra ❑ Lundby ❑ Majorna

❑ Olskroken ❑ Sannegården ❑ Selma

❑ Styrsö ❑ Topas ❑ Torslanda

❑ Tuve

1. How much do your teeth impact your life?

Please check the appropriate box.

❑ ❑ ❑ ❑ ❑

Not at all A little Neither A lot Very much

little nor a lot

1. In the past 3 months, how often have you experienced toothache?

Please check the appropriate box.

❑ ❑ ❑ ❑

Never Once/ Once/ Daily

month week

1. In the past 3 months, how often have you been bothered by food getting stuck between your teeth?

Please check the appropriate box.

❑ ❑ ❑ ❑

Never Once/ Once/ Daily

month week

1. In the past 3 months, how often have you experienced meals taking unnecessarily long to eat?

Please check the appropriate box.

❑ ❑ ❑ ❑

Never Once/ Once/ Daily

month week

1. In the past 3 months, how often have you had difficulty biting with your teeth?

Please check the appropriate box.

❑ ❑ ❑ ❑

Never Once/ Once/ Daily

month week

1. In the past 3 months, how often have you had difficulty chewing?

Please check the appropriate box.

❑ ❑ ❑ ❑

Never Once/ Once/ Daily

month week

1. In the past 3 months, how often have you had difficulty pronouncing certain words or letters?

Please check the appropriate box.

❑ ❑ ❑ ❑

Never Once/ Once/ Daily

month week

1. In the past 3 months, how often have you felt embarrassed due to your teeth?

Please check the appropriate box.

❑ ❑ ❑ ❑

Never Once/ Once/ Daily

month week

1. In the past 3 months, how often have you been concerned about what other people think because of your teeth?

Please check the appropriate box.

❑ ❑ ❑ ❑

Never Once/ Once/ Daily

month week

1. In the past 3 months, how often have you avoided smiling or laughing because of your teeth?

Please check the appropriate box.

❑ ❑ ❑ ❑

Never Once/ Once/ Daily

month week

**14)** In the past 3 months, how often have you been teased because of your teeth?

Please check the appropriate box.

❑ ❑ ❑ ❑

Never Once/ Once/ Daily

month week

1. In the past 3 months, how often have others questioned or commented about your teeth?

Please check the appropriate box.

❑ ❑ ❑ ❑

Never Once/ Once/ Daily

month week

**16)** In the past 3 months, how often have you felt unwell because of the positioning of your teeth?

Please check the appropriate box.

❑ ❑ ❑ ❑

Never Once/ Once/ Daily

month week

1. I want braces. ❑ Yes ❑ No

**18)** I want braces because:

Please check the boxes that apply to you.

❑ ❑ ❑ ❑ ❑

To feel better Improve Be able to smile/ Get a better Feel

overall appearance laugh without job in the self-assured

inhibition future

❑ ❑ ❑

Invest in my oral health Easier to socialize with others My parents think so

❑ ❑

Society expects straight teeth Easier to attract a girlfriend/boyfriend

❑ It’s free ❑ Avoid future regrets ❑ I don’t want braces

❑ Another reason…………………………..

**19)** How significant do you feel your need for braces is?

Please check the appropriate box.

❑ ❑ ❑ ❑

None Small Great Very great

**20)** Who was the first person to suggest that you need braces?

Please check the boxes that apply to you.

❑ ❑ ❑ ❑ ❑

Myself Family Peers My girlfriend/boyfriend Dentist

**21)** How much would you be willing to pay for braces if the treatment was not free of charge?

Please check the appropriate box.

❑ ❑ ❑ ❑ ❑

<5000kr 5000 - 10000 - 20000 - >30000kr

10000kr 20000kr 30000
